# TOTAL COST OF HOSPITALIZATION OF PATIENTS UNDERGOING ELECTIVE LAPAROSCOPIC CHOLECYSTECTOMY RELATED TO NUTRITIONAL STATUS

**DOI:** 10.1590/0102-6720201600020004

**Published:** 2016

**Authors:** Francisco Julimar Correia de MENEZES, Lara Gadelha Luna de MENEZES, Guilherme Pinheiro Ferreira da SILVA, Antônio Aldo MELO-FILHO, Daniel Hardy MELO, Carlos Antonio Bruno da SILVA

**Affiliations:** 1 Master Degree in Public Health Program, University of Fortaleza; 2 Dr. Waldemar de Alcântara General Hospital, Fortaleza, Ceará, Brazil

**Keywords:** Cost control, health expenditures, Cholecistectomy, Obesity, Laparoscopy

## Abstract

**Background:**

In the Western world, the population developed an overweight profile. The morbidly obese generate higher cost to the health system. However, there is a gap in this approach with regard to individuals above the eutrofic pattern, who are not considered as morbidly obese.

**Aim:**

To correlate nutritional status according to BMI with the costs of laparoscopic cholecystectomy in a public hospital.

**Method:**

Data were collected from medical records about: nutritional risk assessment, nutricional state and hospital cost in patients undergoing elective laparoscopic cholecystectomy.

**Results:**

Were enrolled 814 procedures. Average age was 39.15 (±12.16) years; 47 subjects (78.3%) were women. The cost was on average R$ 6,167.32 (±1830.85) to 4.06 (±2.76) days of hospitalization; 41 (68.4%) presented some degree of overweight; mean BMI was 28.07 (±5.41) kg/m²; six (10%) individuals presented nutritional risk ≥3. There was a weak correlation (r=0.2) and not significant (p <0.08) between the cost of hospitalization of the sample and length of stay; however, in individuals with normal BMI, the correlation was strong (r=0,57) and significant (p<0.01).

**Conclusion:**

Overweight showed no correlation between cost and length of stay. However, overweight individuals had higher cost of hospitalization than those who had no complications, but with no correlation with nutritional status. Compared to those with normal BMI, there was a strong and statistically significant correlation with the cost of hospital stay, stressing that there is normal distribution involving adequate nutritional status and success of the surgical procedure with the consequent impact on the cost of hospitalization.

## RESUMO

**Racional:**

No mundo ocidental, a população desenvolveu um perfil de excesso de peso corporal. Os obesos mórbidos geram custo mais alto para o sistema de saúde. Entretanto, observa-se um hiato no tocante aos indivíduos acima do eutrofismo, mas não considerados obesos mórbidos.

**Objetivo:**

Correlacionar estado nutricional, segundo o IMC, com custo de internação de colecistectomias videolaparoscópicas.

**Método:**

Coleta de dados dos prontuários sobre: avaliação de risco nutricional, estado nutricional e custo de internação de pacientes submetidos à colecistectomia videolaparoscópica eletiva no período de janeiro de 2012 a dezembro de 2014.

**Resultados:**

Foram analisados 814 procedimentos. A idade média foi de 39,15 (±12,16) anos; 47 (78,3%) eram mulheres. O custo de internação foi, em média, de R$ 6.167,32 (±1.830,85) para 4,06 (±2,76) dias de internação. Quarenta e um (68,4%) pacientes apresentavam algum grau de sobrepeso; o IMC médio foi 28,07 (±5,41) kg/m²; seis (10%) indivíduos apresentavam risco nutricional ≥3. Houve correlação fraca (r=0,2) e não significativa (p<0,08) entre o custo de hospitalização e o tempo de permanência. No entanto, em indivíduos com IMC normal, a correlação foi forte (r=0,57) e significante (p<0,01).

**Conclusão:**

Sobrepeso não demonstrou correlação entre custo e tempo de internação. Entretanto, os indivíduos com sobrepeso apresentaram custo maior de internação em relação aos que não tiveram intercorrência, mas sem correlação com o estado nutricional. Em relação aos com IMC normal, houve correlação forte e estatisticamente significante com o custo para tempo de internação, reforçando que há provável distribuição normal envolvendo estado nutricional adequado e sucesso do procedimento cirúrgico com consequente impacto no custo de internação.

## INTRODUCTION

In the current globalized and connected world, the rational use of available resources, natural or financial, is the agenda of numerous discussions. In health, controversies follow the same line. In Brazil, it is not different, due to the Unified Health System (SUS), which recognizes health as a right of all citizens and a duty of the State.

 The total cost of hospitalization is the sum of several factors such as those related to diagnostic services and therapy, including complementary examinations and surgical procedures. In surgery, it may occur after performing elective/emergency surgery, which may cause variations in costs for the same surgical procedure^13^. The one which may fit in such conditions is cholecystectomy. In 2013 in the city of Fortaleza, Ceará, Brazil were allocated R$ 2,682,666.88 with admissions due to cholelithiasis and cholecystitis, representing 8.35% of hospitalization costs with benign diseases of the digestive system for the city in that period. Among hospitalizations for cholelithiasis 73.68% were for elective hospitalization^5^.

 Cholecystectomy is considered a safe surgical procedure, and presents mortality rate of 0.1% in patients under the age 50 and 0.5% above. Morbidity can vary from 3-5%^4^, and is related to infectious and non-infectious complications; longer hospitalization is also considered as a complication^2^.

 As obesity is an "epidemic plague" in the United States^17^ and in Brazil^3^, suggesting that more people can be overweight (pre-obese and obese) when undergoing surgical procedures. Obesity is a co-morbidity risk factor that increases health care costs^11^ ^,^ ^12^ ^,^ ^17^.

Due to adipose tissue is relatively avascular - leading to poor tissue perfusion -, the overweight individuals may develop more complications when in surgery^16^ ^,^ ^17^, and higher cost in hospitalization.

The objective of this study was to evaluate the total cost of hospitalization of patients undergoing elective laparoscopic cholecystectomy in a public hospital correlated with the nutritional status.

## METHOD

This study was submitted to the Research Ethics Committee of the University of Fortaleza, Brazil, being authorized under n^o^. 957 785. It was held at Dr. Waldemar Alcântara General Hospital located in Fortaleza, Ceará, Brazil from primary data contained in hospital records retrospectively. Data collection was performed in the first half of 2015 using the medical file services of the public health system hospitals. The participants were patients hospitalized for elective laparoscopic cholecystectomy in the period of January 1^st^ 2013 to December 31^st^ 2014, subject to the following inclusion criteria: age 18-59 years and with nutritional assessment described in medical records.

Pregnant women, nursing mothers, bedridden, terminally ill and patients with infection prior to the surgical procedure, malignancy or inflammatory bowel disease, were excluded.

 The cost of admission was obtained from the hospital files, containing hospital daily needs, surgical procedures and hospitalization values. 

Nutritional status was obtained by the BMI recorded in patients who had hospitalization longer than 48 h. BMI was assessed according to the World Health Organization classification in 1997^18^. Also, the National Risk Score 2002 in individuals under 60 years was collected. Screening of nutritional risk is an instrument that produces a final score from the sum of scores in two columns - one for loss of nutritional status and the other for disease severity - having a significant correlation with the clinical course of patient's hospital stay^14^.

Sociodemographic data (gender, age), surgical comorbidities, perioperative complications, and/or postoperative complications, time spent in the operating room, were also collected to characterize the sample and determine factors that may have affected the hospitalization cost.

### Statistical analysis

Data were tabulated and analyzed by the Statistical Package for Social Sciences (SPSS for Windows, version 20). The results for the socio-demographic data, surgical clinical aspects related to hospitalization and nutrition were presented as descriptive statistics. For the statistical analysis, was used the Pearson correlation coefficient (r), Student's t test for independent samples and chi-square (x ²). p value <0.05 was considered statistically significant.

## RESULTS

**FIGURE 1:**
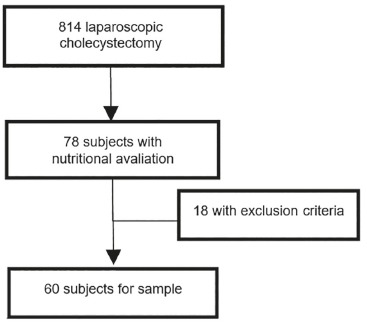
Sample Design

Figure 1 describes the selection of the sample, 18 individuals were taken out due to exclusion criteria.

Of the 60 included, 47 (78.33%) were women. The average age was 39.15+12.16 years. The average length of stay in the operating room, including all surgical and anesthetic procedures, was 155.57±67.33 min. Of all, 18 (30%) had associated disease, being systemic hypertension the most prevalent (15%). Thirteen (21.7%) had a previous episode of acute cholecystitis not related to hospitalization and eight (13.3%) had history of pancreatitis. Prior to cholecystectomy, surgical procedures occurred in 14 (23.3%), being caesarean (8), appendectomy (2), inguinal hernia (2) and abdominal hysterectomy (2). The overall rate of postoperative complications was 2.46%; paralytic ileus was the most common complication, occurring in nine (15%) individuals. Other reported complications were hematoma/dehiscence in the surgical wound in three (5%) patients and biliary fistula in three (5%). Other less common complications were pulmonary infection, atelectasis, pancreatitis, intra-abdominal collection and sepsis. There were four (6.7%) conversion to laparotomy; six (10%) biliary lesions; six (10%) drain settings due to bleeding and biliary leakage; one (1.6%) intestinal lesion; and four had other complications.

To calculate the average stay length and total cost of hospitalization, one patient was excluded due to iatrogenic injury in the main bile duct staying hospitalized for 167 days, the longest period. The average total cost of hospitalization (n=59) was R$ 6,167.32±1,830.85; the average length of hospital stay was 4.06±2.76.

The mean BMI was 28.07±5.41 kg/m². In relation to the World Health Organization classification for nutritional status according to BMI, 41 (68.4%) were overweight.

**FIGURE 2:**
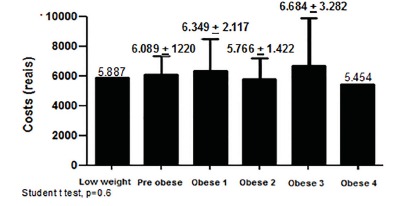
Average hospitalization value (in R$) related to nutritional status by BMI (WHO, 1997)

 The assessment of nutritional risk by NRS 2002 was performed in 42 (70%) patients, of whom six (10%) were at nutritional risk. The average total cost of hospitalization tended to rise, following the increase of the BMI classification of nutritional status (Figure 2), although not statistically significant (p=0.6).

The average total cost of hospitalization showed no statistic significant difference (p=0.8) when comparing the overweight group and normal weight.

There was a significant difference between with and without intra-operative complications (7,204.00+/-1,986.00 vs. 5,631.00+/-1,494.00, p<0.001).

Among individuals who developed complications, there were no significant cost differences (p=0.6).

**FIGURE 3:**
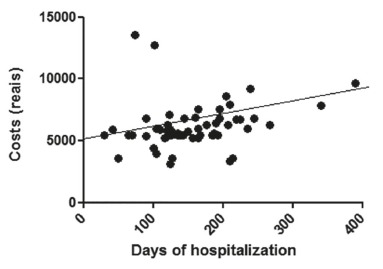
Correlation between cost of hospitalization and hospital stay (in hours)

**FIGURE 4:**
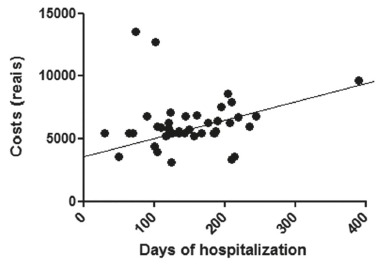
Correlation between cost of hospitalization and hospital stay (in hours) in overweight BMI

**FIGURE 5:**
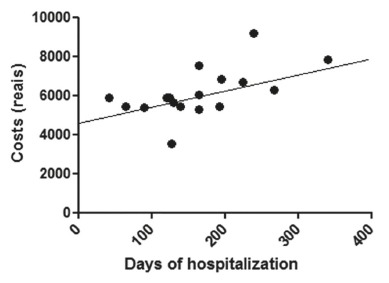
Correlation between cost of hospitalization and hospital stay (in hours) for individuals with normal BMI

Figures 3, 4 and 5 show the correlations between the costs and the hospitalization period (in hours) for the sample and for subgroups of BMI overweight and normal.

## DISCUSSION

The mean time in the operating room was more than 2 h. Such permanence refers to anesthesia induction, surgical procedure, awakening the patient and putting him out of the operating room to post-anesthetic recovery room, not only the surgery in itself. This time also allowed observe postoperative events still in the operating room, such as respiratory failure after extubation, frequent in obese people, needing a second intubation.

Hypertension was the most common co-morbidity. It is estimated that 60% of hypertensive patients are more than 20% overweight.

Surgical procedures previously performed were below the mesocolon, not causing adherence to the surgical site of the gallbladder. Operative procedures that result in adhesions at the surgical site may become a risk factor for conversion or postoperative complications in laparoscopic cholecystectomy, generating a direct impact on time of hospital stay^7^.

The cholecystitis and pancreatitis by producing adhesions at the surgical site or the performance of endoscopic retrograde cholangiopancreatography for calculus disease treatment in the main bile duct, are considered risk factors for conversion, increased surgical time and biliary fistula, directly impacting the hospital stay and, consequently resulting in higher costs for health care system^15^.

The studies that reported complications related to laparoscopic cholecystectomy tend to consider separately the conversion and other complications^6^ ^,^ ^7^ ^,^ ^9^ ^,^ ^15^ ^,^ ^19^. However, some situations that occur during the perioperative are not included in the ratings of postoperative complications^2^. Similar study conducted in Eastern Europe^9^ refers complications, but without mentioning events during surgery, such as drainage or intestinal injury. In this study, these events were called intercurrences. Drainage due to bleeding or bile leakage in the surgical bed, and the injury of common bile duct were the most frequent complications. Conversion to laparotomy was based on adhesions, uncontrolled bleeding in the laparoscopic technique and biliary injury^6^ ^,^ ^9^.

The complications in the postoperative period were equally divided between infectious and non-infectious. Among non-infectious, paralytic ileus was responsible for the vast majority, accounting for 9/10 cases. The overall complication rate for laparoscopic cholecystectomy in this study (2.46%) is similar to that reported in literature, which can range from 0.5-3.0%^15^.

 Postoperative complications and intra-operative complications were not correlated with overweight, even separated in different overweight levels adopted by the World Health Organization^18^. Similarly, there was no correlation between gender and postoperative complications or intercurrences.

The overweight person, even morbidly obese, cannot be considered at risk for conversion or postoperative complication and should be considered in the same category as those with normal weight for laparoscopic cholecystectomy, following the same routine in postoperative^7^.

Regarding hospitalization costs, the group with postoperative complications was not significantly different compared to those who did not develop complications. Individuals who have had complications during surgery, in turn, had higher costs of hospitalization compared with those without complications. In this group of patients a prolonged hospitalization was necessary, which may not occur in cases of post-surgery complication^4^ ^,^ ^8^.

 The average length of stay for this sample was four days, which is above the one described in the literature. According to the normative regulation 131/2006 of the National Agency of Health Insurance, the amount paid for hospital admission (AIH) for performing laparoscopic cholecystectomy (SUS code 33015082) was R$ 601.69, corresponding to surgical and two days of hospital stay^19^. The average cost of hospitalization of this sample was about 10 times that amount. Probably this high cost was due to the individuals who had complications and intercurrences. When considering the minimum cost of the sample, it is approximately five times the predicted value in the SUS payment, showing large discrepancy between these values. 

Another factor associated with this very high cost is that the hospital where this research was conducted is educational for the medical residency program in general surgery. Even with the presence of a medical supervisor and, where medical supervisor is the main surgeon, laparoscopy is dependent on a well-trained and experienced professional staff, especially if considered an emergency environment^6^, which was not the case in this study, because all surgical procedures were elective.

In this series there was prevalence in overweight - 65% of patients were in some degree of being overweight. However, excess weight did not cause impact on the cost of hospitalization. It is noteworthy, however, that there was a trend of increased cost of hospitalization as the classification of nutritional status by BMI moves to higher overweight levels^14^.

The elderly over 60 years, because they have peculiar characteristics as the nutritional status and food intake, were excluded from the study. 

 In this series, was identified weak correlation without statistical significance between cost and length of stay. Result on costs certainly is dependent on the length of hospital stay, but not limited to this factor. For example, a factor that may contribute to the costs, without necessarily increasing hospital stay, is the need for intensive support in the immediate postoperative period.

Weak correlation with no statistical significance was found between the costs and the BMI overweight. Although the sample was mostly composed by obese, overweight was not responsible by elevated costs^3^ ^,^ ^17^. This fact was also reported in an American study with over a thousand patients where high BMI was not identified as a factor associated with postoperative complications, longer hospital stays and, therefore, higher cost^7^. Peculiarly and in interesting way, this study showed strong correlation between the cost of hospitalization and length of stay for individuals with normal BMI. This correlation is supported by evidence of individuals in adequate nutritional status have fewer surgical site infections than individuals with low weight or obesity^2^.

Diagnosis of adequate nutritional status, BMI within the normal range of biochemical markers of nutritional status within normal parameters should be viewed positively and should raise the multidisciplinary team effort to maintain these findings, since individuals outside this normal interval tend to develop more postoperative complications and have longer hospital stays and higher hospital stay costs^10^. The result of the correlation between normal BMI and hospital costs also require larger sample or prospective study to confirm this correlation. 

## CONCLUSION 

Overweight showed no correlation between cost and length of stay. However, overweight individuals had higher cost of hospitalization than those who had no complications, but with no correlation with nutritional status. Compared to those with normal BMI, there was a strong and statistically significant correlation with the cost of hospital stay, stressing that there is normal distribution involving adequate nutritional status and success of the surgical procedure with the consequent impact on the cost of hospitalization.
